# Supplementary material for: Are carbon emissions trading and green financial instruments synergistic? -Comprehensive quantitative research based on content analysis
Source: PLoS One. 2024 Mar 7;19(3):e0298601. doi: 10.1371/journal.pone.0298601 (PMC10919601; doi:10.1371/journal.pone.0298601)
Supplement: S1 Data — (ZIP) [file pone.0298601.s001.zip › Data/地级市Z_F工作报告.zip!/╣≤╓▌/┴∙┼╠╦«╩╨/└┤╫╘╣1⁄2╓┌║┼í╛┬φ┐╦╩2╛▌═°í┐_2017.docx]

各位代表：

　　现在，我代表市人民政府向大会作工作报告，请予审议，并请市政协委员和其他列席人员提出意见。

　　一、过去五年工作回顾

　　过去五年，是我市经受住各种困难和风险考验，在全面建设小康社会进程中取得新成就的五年。在市委的坚强领导下，在市人大、市政协的监督和支持下，我们深入学习贯彻习近平总书记系列重要讲话精神特别是对贵州工作的重要指示精神，统筹推进“五位一体”总体布局，协调推进“四个全面”战略布局，自觉践行五大发展理念，始终坚持主基调主战略，牢牢守住发展、生态、安全三条底线，全力以赴稳增长、促改革、调结构、惠民生、防风险，完成了市七届人大历次会议确定的主要任务。

　　地区生产总值达到1313.7亿元、年均增长14%，人均生产总值达到45325元；一般公共预算收入达到133.69亿元，年均增长13.6%；固定资产投资累计完成4624.14亿元，年均增长26.1%；金融机构人民币存贷款余额分别达到1207.75亿元、935.56亿元，年均增长15.9%和17.5%。盘县进入全国百强县、西部十强县，位次逐年前移；钟山区、水城县、六枝特区跻身全省经济强县行列。预计减少贫困人口65.87万人，贫困发生率从2011年的38.3%下降到11.91%。全面小康实现程度预计达到84.6%，年均提高4个百分点，盘县、钟山区被认定为全省同步小康创建达标县。

　　五年来，我们聚焦聚力脱贫攻坚头等大事，精准扶贫卓有成效。深入实施大扶贫战略行动，作出“决战三年、摆脱贫困”决策部署，制定“33669”脱贫攻坚行动计划，推进“216”开放式扶贫试验区建设，实现68个贫困乡镇摘帽。全国产业精准扶贫现场观摩交流总结会在我市召开。全力打好易地扶贫搬迁“当头炮”，实施易地扶贫搬迁1.44万户5.62万人，实现搬迁移民稳定就业1.16万人。建立“五定五看”精准扶贫评价体系，建成大数据“扶贫云”平台。压缩6%的行政经费用于教育精准扶贫。创新扶贫机制，探索建立“211”集团帮扶模式，政府、市场和社会协同推进的大扶贫格局正在形成。

　　五年来，我们聚焦聚力转型升级主攻方向，经济结构持续优化。深入推进供给侧结构性改革，促进一产转型、二产升级、三产优化。“5个100工程”累计完成投资2580.71亿元。三次产业结构从2011年的5.2∶62.7∶32.1调整为2016年的9.6∶50.2∶40.2。大力发展山地特色农业，获批创建六盘水国家农业科技园区和全国生态原产地产品保护示范城市，水城县获批创建国家有机产品认证示范区；大力实施“3155工程”，粮经比由70∶30调整为32∶68；新增省级农业园区29个，市级以上农业龙头企业89家、省级龙头企业45家；成功打造凉都“弥你红”“天刺力”等知名农产品品牌，获得“三品一标”产地认定、产品认证证书308个，国家地理标志保护产品21个；建成4个县级电子商务服务运营中心、519个农村电子商务服务站点。大力推进工业转型升级，获批创建国家循环经济示范城市；实施煤炭产业“四个一批”，煤炭资源就地转化率达到80%，煤矿机械化开采率达到69%；推进煤气共采一体化发展，煤层气综合利用率达到43%。大数据、大健康、新型建筑建材等新兴产业发展风生水起，产值达到263亿元。大力发展以旅游业为龙头的现代服务业，全力打造“中国凉都”山地旅游升级版，成功承办省第八届旅游产业发展大会，连续举办三届全市旅游文化产业发展大会，冬季滑雪、康体养身、山地运动、农旅文融合等旅游新业态从无到有、势头强劲，建成国家4A级景区3个、3A级景区14个、省级旅游度假区3个，接待游客人次和旅游总收入分别年均增长41.9%、39.1%，实现“井喷式”增长。大力实施“引金入市”工程，引进银行、保险和证券机构9家，设立产业基金3支。新增新三板挂牌企业1家。钟山区获批国家级服务业综合改革试点。

　　五年来，我们聚焦聚力基础设施建设重点，发展基础不断夯实。以交通水利为重点的基础设施建设取得历史性突破，沪昆高铁境内段建成通车，安六城际铁路加快建设；建成高速公路6条，通车里程达到380公里，成为全省第二个县县通高速的市州；完成国省道改造107公里、建成通村油（水泥）路5933公里，行政村通畅率达到98.5%；月照机场通达15个城市，盘州官山机场完成选址工作。建成中型水库5座、小型水库13座，实现每个县有1座以上中型水库目标，工程性缺水问题和农村人口饮水安全问题得到有效缓解。加快完善电力通信基础设施，建成500千伏、220千伏、110千伏变电站29座，新增线路7631公里，农村电网网改率达到100%。实现“乡乡通宽带”“村村通电话”，重点旅游景区、星级酒店WIFI全覆盖。

　　五年来，我们聚焦聚力城乡一体重要抓手，区域发展协调推进。坚持产城景互动共融，走山地特色新型城镇化道路，按照“四域统筹”“八规合一”，加快两个“三地同城化”发展，全市常住人口城镇化率达到49.5%。建成内环快线、机场高速等城市主干道路，地下综合管廊、水城河综合治理等重大工程加快推进，建成6个城市湿地公园等公共服务设施，城市承载能力不断提升。六枝至水城天然气支线管道建成投用，全面完成市中心城区天然气置换工程，市民告别“望气兴叹”的历史。实施市中心城区城市山体保护和景观提升，城市人均公园绿地面积达到10.61平方米。深入开展“四创”活动，城市精细化管理水平持续提高。成功承办第五届全省小城镇建设发展大会，打造34个特色小城镇，六枝特区郎岱镇入选中国特色小镇。六个小康行动计划累计完成投资131.4亿元。建成美丽乡村省级示范点21个、省级少数民族特色村寨51个，9个村落进入“中国传统村落”名录。盘县获批“全国第三批新型城镇化试点县”“全国美丽乡村建设标准化试点县”。

　　五年来，我们聚焦聚力生态文明战略任务，绿色理念深入人心。坚持以生态产业化、产业生态化为引领，加快推动绿色发展。大力实施“绿色贵州”建设三年行动计划，完成营造林面积336.59万亩，治理石漠化301.7平方公里、水土流失1363.04平方公里，森林覆盖率达到52.77%。创建省级生态乡镇8个、省级生态村24个。大力实施环境污染治理设施建设三年行动计划和“十二件环保实事”，建成19个城镇污水处理厂，城镇污水处理率、城乡生活垃圾无害化处理率分别达到86%和81.06%，全市空气质量优良率达到96.4%。单位生产总值能耗累计下降28.66%。钟山区获批全国农村生活污水治理示范区。制定实施《六盘水市水城河保护条例》。认真落实“大气十条”“水十条”“土十条”，严格执行“六个严禁”“六个一律”，始终做到铁腕治污，严肃查处环保违法行为。

　　五年来，我们聚焦聚力改革开放关键一招，动力活力显著增强。谋划重点改革专题292个，出台方案252个。“三变”改革成为全省农村改革示范样板并在全国推广，农村产权确权登记颁证稳步推进，建成市级和3个县级农村产权交易中心。深化“放管服”改革，取消行政审批事项113项，下放审批事项134项。国有企业、财税金融、教育卫生、司法体制、社会治理等重点领域改革有序推进。大力实施提高民营经济比重五年行动计划，民营经济增加值占比从2011年的40.43%提高到56.54%。六盘水海关开关运行。积极融入国家“一带一路”和长江经济带，成功举办生态文明贵阳国际论坛六盘水分论坛、中国—东盟国际产能合作妥乐论坛等主题论坛。引进500强企业27家，累计完成招商引资省外到位资金3075.27亿元。实际利用外资年均增长59.73%。深入实施大众创业、万众创新，综合科技进步水平指数达到46.9%。

　　五年来，我们聚焦聚力民生福祉根本目的，发展成果更加惠民。坚持每年启动实施一批民生工程和民生实事，累计实施民生实事项目376个，完成投资420.5亿元。完善就业服务体系，累计新增城镇就业39.7万人次，城镇、农村居民人均可支配收入年均分别增长9.9%和12.8%，城乡居民收入比从3.53∶1下降到3.1∶1。完成教育“9+3”计划，基本建成高中教育城、以朵职教城等重大项目，实现公办幼儿园乡镇全覆盖、营养改善计划农村义务教育阶段学校全覆盖，六枝特区、盘县、钟山区通过省“普十五”验收，六盘水师范学院通过教育部本科教学评估验收。全面推开县级公立医院改革，启动城市公立医院改革，创建三级医院5家，基本建成凉都医院，实现“五个全覆盖”目标。认真落实全面两孩政策。深入实施文化惠民工程，建成贵州“三线”建设博物馆、凉都体育中心、凉都大剧院等文化体育设施，连续举办凉都消夏文化节，凉都夏季国际马拉松上升为国家金牌赛事，山地运动水平不断提升，影响力显著增强。社会保障水平持续提高，城乡低保标准分别提高到545元/月、3132元/年。实施农村危房改造15.9万户、棚户区改造20万户64万人、保障性住房4.3万套。加强农村留守儿童、困境儿童、留守老人精准关爱救助保障。深入推进“2221”安全生产体系建设，盘县、水城县从全国50个煤矿安全重点县中脱帽，安全生产形势持续向好。获批创建国家食品安全示范城市。深化“平安凉都”建设，群众安全感、满意度逐年提高。国防建设与经济建设协调发展。民族、宗教、外事、侨务、对台、人防、档案、妇女、儿童、老龄、残疾人、地方志等各项事业取得新进步。

　　五年来，我们聚焦聚力自身建设基本要求，政府职能加快转变。深入开展党的群众路线教育实践活动、“三严三实”专题教育和“两学一做”学习教育，坚持不懈反“四风”、转作风、促政风。公布35个市级行政机关行政权力清单和责任清单。市县两级审批服务系统全部迁入省“电子政务云”，政务服务事项提前办结率达到90%，办事群众满意率达到96%。全面推行政府性投资项目前置审计。强化督查督办，构建抓执行落实的任务链责任链。大兴调查研究之风，着力研究问题、破解难题。推进政府系统全面从严治党主体责任落实，强化行政监察和审计监督，深入开展民生监督，勤政廉政建设取得新成绩。

　　刚刚过去的2016年，我们坚持以“开局就是决战、起步就是冲刺”的劲头，沉着应对经济下行压力，强化运行调度和精准服务，全市经济发展稳中提质，实现“十三五”良好开局。地区生产总值增长12%；规模以上工业增加值增长10%，固定资产投资增长22%，社会消费品零售总额增长13.3%，金融机构存贷款余额分别增长23.64%和19.98%。“十件民生实事”基本完成。

　　各位代表，过去五年是我市发展进程中极不平凡的五年，成绩来之不易，经验弥足珍贵。五年的历程启示我们，必须把“四个意识”作为自觉行动，坚决同以习近平同志为核心的党中央保持高度一致，确保中央和省各项决策部署在六盘水落地生根、开花结果；必须把善谋善为作为基本能力，运用五大发展理念引领经济社会发展，吃透上情、把握市情、了解外情，推动弯道取直、后发赶超；必须把加快发展作为第一要务，全面贯彻稳中求进工作总基调，在加快发展中决战脱贫攻坚、助推转型升级、同步全面小康；必须把改革开放作为强大动力，以改革促进开放、以开放倒逼改革，持续释放新的动力活力，为加快发展注入勃勃生机；必须把真抓实干作为根本要求，牢固树立实干兴邦的思想，撸起袖子加油干，迈开步子加快赶，用实干推动新跨越、创造新辉煌。

　　过去五年的成绩，是省委、省政府和市委坚强领导的结果，是市人大、市政协监督支持的结果，是全市各族干部群众团结奋斗的结果。在此，我代表市人民政府，向全市各族人民、各级干部表示崇高的敬意！向人大代表、政协委员，向各民主党派、工商联、无党派人士、人民团体，向驻市人民解放军、武警官兵和人民警察，向所有关心和支持我市改革发展的各界人士，表示衷心的感谢！

　　在肯定成绩的同时，我们必须清醒地看到存在的困难和问题。主要是：调结构、促转型任务艰巨，传统产业偏单偏重，农业“接二连三”融合度不高，现代服务业发展质量和效益还有待提升。抓统筹、奔小康任务艰巨，城乡二元结构、城市二元结构、农村二元结构矛盾较为突出，脱贫攻坚任务十分繁重。优生态、护环境任务艰巨，生态建设欠账多，生态环境脆弱，水土流失和石漠化治理难度大，节能减排面临较大压力。保民生、促和谐任务艰巨，公共服务供给不足，教育、医疗卫生等社会事业不能有效满足群众需求，安全生产基础保障能力依然薄弱。转作风、提效能任务艰巨，少数干部不敢担当、不愿作为，违纪违法现象时有发生，从严治政还需进一步加强。对存在的问题，我们将采取有效措施，认真加以解决。

　　二、今后五年目标任务

　　站在新的历史起点上，我们迎来了经济发展新机遇，进入了转型升级新阶段，开启了全面小康新征程。以习近平同志为核心的党中央提出的治国理政新理念新思想新战略，为我们指明了前进方向、注入了强大动力、提供了根本遵循；省委赋予六盘水统筹城乡改革和发展先行市的新坐标新定位新使命，为我们明确了奋斗目标、规划了战略路径；市第七次党代会以协调推进“四个全面”、统筹城乡改革发展为主题，提出了“五大目标”和“七个新跨越”发展任务，描绘了六盘水今后五年发展的宏伟蓝图。我们要更加紧密地团结在以习近平同志为核心的党中央周围，更加坚定地维护以习近平同志为核心的党中央权威，认真贯彻落实党中央、国务院和省委、省政府及市委的决策部署，保持昂扬斗志，树立必胜信心，按下快进键、跑出加速度、开创新局面，全面建成更高质量、更高水平、更可持续的小康社会！

　　今后五年政府工作的总体要求是：高举中国特色社会主义伟大旗帜，坚持以马克思列宁主义、毛泽东思想、邓小平理论、“三个代表”重要思想、科学发展观为指导，深入贯彻习近平总书记系列重要讲话精神，全面贯彻落实党的十八大和十八届三中、四中、五中、六中全会及省委十一届八次全会、市第七次党代会精神，统筹推进“五位一体”总体布局和协调推进“四个全面”战略布局，坚持稳中求进工作总基调，贯彻落实五大发展理念，坚持实施主基调主战略，推进“三大战略行动”，守住“三条底线”，抓好“三件大事”，走好“四条新路”，弘扬“三线”精神，深化“三变”改革，全面建设统筹城乡改革和发展先行市，全力实现六盘水经济社会发展新跨越。

　　今后五年经济社会发展的主要预期目标是：地区生产总值年均增长12%左右；3个国家级扶贫开发重点县，贫困村全部出列，全面消除城乡贫困人口；规模以上工业增加值年均增长10%；固定资产投资年均增长20%左右；社会消费品零售总额年均增长12%；一般公共预算收入年均增长6%左右；城镇、农村居民人均可支配收入年均分别增长11%和12%以上；城镇新增就业44.5万人以上，城镇登记失业率控制在4.2%以内；服务业比重提高到45%左右；森林覆盖率达到63%以上，完成省下达的节能减排指标任务。通过五年努力，经济社会发展实现“一方阵五前列”。

　　围绕实现上述目标，我们将坚持做到“六个科学统筹”，着力构建“六大体系”，不断开创统筹城乡改革和发展新局面。

　　（一）科学统筹结构调整和转型升级，着力构建优势突出的现代产业体系。坚持以供给侧结构性改革为主线，深入实施“六大工程”，推动一产转型、二产升级、三产优化。实施传统产业提质工程。按照立足煤、做足煤、不唯煤的总体思路，以开放性思维、战略性眼光，大力实施煤电化一体化，实现煤炭产业集团化发展、集约化利用、机械化采掘、循环化发展。到2021年，全市煤矿企业从业人员素质提升达到100%，符合条件的矿井机械化开采达到100%，绿色化发展达到100%，安全生产保障能力提升达到100%，煤气共采一体化达到100%。大力推动以煤制油、煤焦气化为重点的精深煤化工取得突破。建成国家循环经济示范城市，打造一批名副其实的循环经济园区、循环经济行业、循环经济产业、循环经济企业，实现资源循环利用、“变废为宝”。推进园区提质升级，培育壮大园区主导产业和企业集群，提高园区发展水平和综合竞争力。到2021年，获批国家级高新区1个以上，建成1个千亿元园区、3个500亿元园区和4个100亿元园区。坚持高端化、绿色化、集约化，坚决杜绝污染项目、落后产能进入园区。大力实施“双百”工程、“双培育”计划和“125”工程，到2021年，引进企业100家以上、其中500强企业20家以上，新增企业市场主体12万户以上、规模以上工业企业400家以上，分别新增引领型、培育型、成长型小微企业100户、200户、500户以上，提高传统工业优质有效供给能力。实施能源产业创新工程。发挥煤炭资源比较优势和竞争优势，加快推进煤电体制机制改革，建立煤电联动、水火互济的能源保障体系，推动能源工业稳定安全可持续发展，打造跨区域能源枢纽。大力发展非常规天然气、地热等清洁能源和新能源，加快光伏发电、垃圾发电发展步伐，形成多能互补、低碳绿色的综合能源体系，以能源结构优化促进传统工业转型升级。实施新兴产业倍增工程。围绕发展数字经济、旅游经济、绿色经济和县域经济，深度融入国家大数据综合试验区、内陆开放型经济试验区和生态文明试验区，加快建设凉都大数据中心、贵州省大数据应用示范基地、贵州西部智能终端产品制造基地，大力发展新材料、生物医药、现代装备制造、天然饮用水等新兴产业，培育新的经济增长极。到2021年，全市新兴产业规模以上工业总产值达到800亿元以上，培育上市企业10户。实施特色农业增效工程。按照强龙头、创品牌、带农户的总体思路，坚持以“3155工程”为载体，以“三变”改革为动力，加快发展猕猴桃、刺梨、茶叶、软籽石榴、小黄姜和区域精品水果等特色产业，巩固发展烤烟产业，加强基本烟田规划建设，大力发展生态畜牧业和农产品加工业，让绿色优质农产品给老百姓带来更多红利。大力培育新型经营主体，推动农业产业“接二连三”，打造全链条、全循环、高质量、高效益的农业产业化集群，建成喀斯特山区特色农业示范区和全国生态原产地产品保护示范城市。实施现代服务业发展工程。顺应大众旅游时代潮流，与时俱进打造山地旅游发展升级版，持续提升“中国凉都”“南国冰雪城”品牌影响力，注重全景式打造、全社会参与、全产业发展、全季节体验、全方位服务、全区域管理。到2021年，接待游客人次和旅游总收入年均增长40%以上，建成1个国家级旅游度假区、1个以上5A级景区、10个以上4A级景区，旅游服务设施和服务功能全面提升，建成西部一流、国内知名的康养产业基地。深入实施“引金入市”工程，构建现代金融服务体系。加快发展现代物流，建成西南地区重要物流节点城市。大力发展电子商务、文化创意等现代服务业。实施“双创”孵化工程。完善科技创新服务和众创空间等创业孵化体系，加大返乡大学生、城乡青年创业创新扶持力度，建设一批“双创”基地，发展一批科技创新型小微企业，培育一批创新型企业家、技术专家和创客团队。

　　（二）科学统筹脱贫攻坚和同步小康，着力构建全面覆盖的民生保障体系。坚持以人民为中心的发展思想，着力补齐城乡短板、缩小城乡差距。打赢精准脱贫攻坚战。按照“四个切实”“六个精准”的总要求，坚持以精准扶贫、精准脱贫为主线，把发展生产扶贫作为主攻方向、把易地搬迁扶贫作为重要补充、把生态补偿扶贫作为双赢之策、把发展教育扶贫作为治本之计、把社会保障兜底扶贫作为基本防线，以“三变”改革为总抓手，深入实施大扶贫战略行动，全力推进“216”开放式扶贫试验区建设，因地制宜探索精准脱贫的有效路子，分类施策、真抓实干，切实做到真脱贫、脱真贫，确保到2018年实现“两不愁、三保障”目标，决不让一个民族、一个村寨、一户群众在脱贫攻坚中掉队，到2020年与全国全省同步全面建成小康社会。推动社会事业上台阶。深入推进教育综合改革，新增省级示范性高中5所、其中一类省级示范性高中2所，新增省级示范性幼儿园10所，实现每个县有2所以上省级示范性高中、1所农民技能培训学校，每个乡镇有1所高标准寄宿制学校、1所标准化公办幼儿园，2018年全面实现“新两基”。把六盘水师范学院办成区域性高水平应用型本科院校，把六盘水职业技术学院办成高级技术技能型人才培养基地。深化医药卫生体制改革，加快推进医疗卫生“十百千”工程，力争每个县有一所以上三甲医院，实现中心乡镇卫生院基本达到二级综合医院水平，六枝特区、盘县、水城县分别建成1所妇女儿童医院。构建文化发展新格局。坚定文化自信，深化文化体制改革，发展文艺事业，壮大文化产业，促进红色文化、历史文化、“三线”文化、民族文化融合发展。加快构建现代公共文化服务体系，大力实施文化惠民工程，市县乡村四级基本公共文化设施覆盖率达到90%以上。织密基本民生保障网。建成国家级创业型城市。扩大城镇职工和城乡居民基本养老、医疗、失业、工伤、生育等社会保险覆盖面。加大棚户区、农村危房改造力度。加快推进城乡社会救助一体化建设，健全社会救助体系，完善特困人员供养、医疗救助、临时救助等制度。筑牢安全稳定压舱石。严格落实安全生产责任制，狠抓企业主体责任、部门监管责任和领导责任落实，狠抓失职追责落实，坚持党政同责、一岗双责，建立安全风险分级管理和隐患排查治理双重体系，夯实安全生产长效基础，从源头上严防重特大公共安全事故发生，实现安全生产形势持续稳定向好。建成国家食品安全示范城市。统筹各项事业发展。重视国防动员和后备力量建设，支持军队、武警部队建设和法院、检察院工作。加强民族、宗教、人防、档案、外事、侨务、对台、妇女、儿童、老龄、残疾人、地方志等工作。

　　（三）科学统筹规划布局和建设管理，着力构建特色鲜明的新型城镇体系。大力推进山地特色新型城镇化建设，到2021年，全市常住人口城镇化率达到56%以上。全力打造中心城市，提升辐射带动能力。遵循城市发展规律，坚持产城景互动、人地钱统筹，围绕两个“三地同城化”，大力实施城市“双修”工程，全力打造以钟山区、双水片区、红桥新区为中心，以南开片区、董地片区为副中心，以六枝特区、盘县为重要城市组团的区域中心城市。坚持市统筹、县为主，支持六枝特区在产城一体上率先突破，盘县在产城景融合发展上率先突破，水城县在生态产业化、产业生态化上率先突破，钟山区在“四域统筹”上率先突破，钟山经济开发区在创新驱动、产城互动上率先突破。全力打造特色小镇，提升融合配套能力。尊重自然格局，整合资源要素，推动新型城镇化与山地旅游、产业发展有机结合，高标准规划建设一批体现山水风光、民族风情、特色风物的特色小城镇。完善小城镇基础设施和配套服务设施，实现建成区供水管网、污水管网、垃圾收运系统全覆盖。到2021年，建成40个以上特色小城镇。全力打造美丽乡村，提升综合发展能力。大力推动农旅文融合发展，深入实施六个小康行动计划，实现“一村一品、一村一景、一村一韵”，打造一批具有生产效益、生活品质、生态价值的美丽乡村。到2021年，打造50个以上美丽乡村升级版示范点，90%以上的村庄完成农村人居环境综合整治。

　　（四）科学统筹集中攻坚和巩固提升，着力构建互联互通的基础设施体系。全面推动交通、水利等基础设施向全域覆盖，不断夯实发展基础。坚持外联内通并重，加快解决交通设施不成网问题。建成安六城际铁路，力争建成盘兴、水盘城际铁路。建成六威、六赫、纳兴、普盘等高速公路，全市高速公路通车里程突破800公里；实施一级公路268公里，普通国道二级及以上公路比例达到90%，普通省道三级及以上公路比例达到70%以上，实现乡镇二级公路全覆盖，全市所有行政村通油路、通客车。实施月照机场改扩建工程，加密月照机场航线，建成盘州官山机场。推进北盘江流域航运建设，建成牂牁江、凉风洞等旅游客运码头。坚持大中小并举，加快解决工程性缺水问题。开工建设1座大型水库、5座中型水库、30座小型水库，实现县县有中型水库、乡乡有小型水库。建成一批农村饮水巩固提升工程，全面解决工程性缺水和农村人口饮水安全问题。新增农田有效灌溉面积30万亩。坚持干线网络并行，加快解决能源信息通道问题。推进新一轮农村电网改造升级，农村地区基本实现稳定可靠的供电服务全覆盖，供电可靠率达到99.8%。统筹推进成品油、天然气管网建设，实现县县通天然气、管道干线乡镇供气全覆盖。深入实施光网城市、“宽带乡村”等工程，建成全光网市，实现行政村“村村通光纤、村村通广电、户户能上网”。

　　（五）科学统筹深化改革和扩大开放，着力构建助推跨越的动力支撑体系。坚持用好改革开放关键一招，持续释放发展动力活力。全面深化改革，增创发展新优势。深化拓展“三变”改革，积极稳妥推进农村集体产权制度改革，推动“三变”改革向城市城镇、工矿企业、政府性投融资公司等领域拓展，全面推进省级“三变”改革试验区建设，争创全国“三变”改革试验区。加快推进行政审批、国企国资、财税金融、司法体制、社会治理等重点领域改革。大力发展民营经济，落实民营经济发展政策措施，构建“亲”“清”新型政商关系，用心用情用力解决发展难题、优化发展环境、减轻发展负担，促进民营企业稳定发展、创新发展、转型发展。到2021年，全市民营经济增加值占比达到60%以上。全面扩大开放，构建发展新格局。深度融入“一带一路”、长江经济带，加强与珠三角、长三角等经济区的合作。实施“走出去”战略，深化与东南亚经贸合作，加强海内外人文交流。用好六盘水海关、中国—东盟国际产能合作妥乐论坛等重大开放平台，建设六盘水保税物流中心、进出口加工区等海关监管区，高水平办好凉都消夏文化节、旅游文化产业发展大会、凉都夏季国际马拉松等竞技体育和山地运动重大活动。大力实施一批引领型外贸项目，加快构建外向型经济体系。继续抓好“5个100工程”重点平台建设。全方位开展招商引资，更加注重招商引资的质量和效益，提高履约率、入园率、资金到位率和投产率，引进市外到位资金年均增长8%。全面加快创新，注入发展新动力。实施人才强市战略，加快引进和培养现代管理人才、企业家人才、高技能人才。鼓励发展创投引导基金，建设国家创新型城市和区域性科技创新中心。大力实施全民科学素质行动计划纲要。到2021年，全市综合科技进步水平指数达到63%以上，高新技术产业产值达到260亿元以上。申报省级创业孵化示范基地5个以上，培育科技企业孵化器4家以上。

　　（六）科学统筹绿水青山和金山银山，着力构建绿色崛起的生态文明体系。坚持生态优先，推动绿色发展，努力实现百姓富与生态美的有机统一。加快发展绿色经济。坚持生态产业化、产业生态化，大力发展“四型十五种”产业，着力构建具有技术含量、就业容量、环境质量的绿色产业体系，提高绿色经济占比，提供更多优质生态产品和服务。加快建造绿色家园。坚持产城景一体、山水田融合、村社园统筹、文体康配套，大力推进山水城市、绿色小镇、美丽乡村、和谐社区建设，实现山水、田园、城镇、乡村各美其美、美美与共。加快完善绿色制度。深化生态文明体制机制改革，健全自然资源资产产权制度、国土空间开发保护制度、资源总量管理和全面节约制度。完善环境监管定责、履责、问责机制，加强环保督察巡查，推动县区环保法庭全覆盖。加快筑牢绿色屏障。大力实施新一轮退耕还林、石漠化综合治理等重点生态工程。坚持以铁的担当治污，加大面源污染治理力度，强化治水、治气、治土、治渣措施，严肃查处生态环保失职渎职、违纪违法行为。加快培育绿色文化。秉承“天人合一、知行合一”的理念，健全生态文明教育体系，广泛开展绿色创建活动，积极倡导绿色生活方式，提高全民绿色意识，引导群众自觉“植绿”“增绿”“爱绿”。

　　三、2017年重点工作

　　今年是实施“十三五”规划的重要一年和推进供给侧结构性改革的深化之年。做好今年各项工作，意义十分重大。

　　今年经济社会发展主要预期目标是：地区生产总值增长12%左右，农业增加值增长7%，规模以上工业增加值增长10.5%，服务业增加值增长14%；减少农村贫困人口10万人；一般公共预算收入增长6%；固定资产投资增长20%；社会消费品零售总额增长13%；金融机构存贷款余额分别增长22%和18%；城镇、农村居民人均可支配收入分别增长11%和12%左右；城镇登记失业率控制在4.2%以内；新增规模以上工业企业100户；森林覆盖率达到55%以上；常住人口自然增长率控制在7‰以内，符合政策生育率达到90%；完成省下达的节能减排指标任务。

　　围绕实现全年目标，我们将重点抓好以下工作：

　　（一）全力以赴抓好大扶贫大数据两大战略行动。深入实施大扶贫战略行动，确保六枝特区按国家标准摘帽，实现184个贫困村出列。扎实推进“216”开放式扶贫试验区建设。制定年度脱贫计划、落实帮扶措施，谋划实施一批脱贫攻坚项目，加快交通水利基础设施建设，全力打好这场超常规战役。扎实推进易地扶贫搬迁工程。精准选好安置点，重点向城镇和园区集中，加强后续扶持和保障，实施易地扶贫搬迁10824户40719人，确保今年3月底前实现2016年第二批搬迁群众入住，2017年项目实现当年入住。扎实推进产业精准脱贫。因地制宜选准主导产业和主攻方向，大力推广“龙头企业+合作社+农户”模式，明确贫困户在产业链、利益链中的环节和份额。加强产品市场开拓，创新产销对接机制，稳定销售渠道。用好管好凉都“三变”产业扶贫基金和财政扶贫资金，提高资金使用效益。

　　深入实施大数据战略行动，推动大数据产业做大做强。编制实施数字经济发展规划，引进一批大数据知名企业，培育壮大一批本土企业，大数据信息产业规模达到100亿元以上。推动大数据与三次产业融合发展。大力实施“互联网+”协同制造专项行动计划，推动煤炭、电力等传统产业提升智能化、自动化水平。加快“三变”大数据信息平台、“三农”信息服务站、食品安全追溯系统等大数据平台建设。推动政府数据聚通用。加快安全云、环保云、医疗健康云、凉都警务云等特色云工程建设并投入运行。引导各行各业依法采集并开放数据。

　　（二）全力以赴抓好供给侧结构性改革。全面落实“三去一降一补”任务。稳步推进煤炭行业化解过剩产能。加快推进棚户区改造和空置商品房消化。继续降低企业融资、物流、税费、制度性交易等成本。深入推进工业供给侧结构性改革。加快国家循环化改造试点建设。积极申报国家老工业城市产业转型升级示范区。推进煤气共采一体化向纵深拓展，正常生产矿井机械化达到78%以上，完成煤层气抽采量13.6亿立方米、利用率达到45%。开展技术改造工程项目100个，引进企业100家以上。加快大健康、新型建筑建材、装备制造等特色优势产业发展。推动园区投产企业稳定发展、建成企业投产达产、落地企业加快建设。深入推进农业供给侧结构性改革。巩固提升“3155工程”，完成补植补种86万亩，推进猕猴桃、刺梨等主导特色产业保险全覆盖。加强农业标准化建设，制定3个特色产业标准化生产体系，建成国家农业科技园区。新增市级以上龙头企业20个以上、农民专业合作社100家以上、家庭农场50个以上。申报国家级出口食品农产品质量安全示范区1个、省级2个，生态原产地保护产品9个，实现“3155工程”无公害产地认证全覆盖。深入推进现代服务业供给侧结构性改革。用好旅游资源大普查成果，编制市县两级全域旅游发展规划。改造提升10个省级景区景点，开工建设六枝至黄果树等5条旅游公路。举办好第四届全市旅游文化产业发展大会。接待游客人次和旅游总收入分别增长40%、50%。加快发展商贸物流、文化体育、会展、健康服务等现代服务业。

　　（三）全力以赴抓好城乡一体化发展。全市常住人口城镇化率提高2个百分点以上。完善城镇规划体系。编制完成红桥、双水、董地联动发展规划和350个村庄规划，实现县域乡村建设规划全覆盖。加强城市建设管理。大力推进棚户区改造。建设地下城市综合管廊18.3公里并完成试点项目建设任务。加快“智慧城市”建设，全力攻坚“四创”，全力争取全国文明城市、国家卫生城市、国家环保模范城市创建成功。整市推进特色小城镇建设。实施全域小城镇建设“3个1工程”，启动5个以上特色小城镇建设，示范小城镇实施“8+X”项目32个，镇村联动覆盖率提高到65%。加快美丽乡村建设步伐。打造20个美丽乡村升级版示范点和20个省级少数民族特色村寨。实施整体改善农村人居环境“10+N”行动计划，启动150个村垃圾治理工程。

　　（四）全力以赴抓好有效投资和消费升级。大力推进项目建设。抓好重大产业项目建设，完成重大工程和重点项目投资1069亿元；完成工业投资425亿元以上，产业投资占比保持在35%。抓好基础设施项目建设，完成交通投资140亿元、水利投资40亿元、电力投资6亿元。加快安六城际铁路及站点、盘兴城际铁路建设，推进六威、六赫高速和大董一级公路建设，开工建设普盘高速、鱼塘至发射高速。启动国省道改造101公里、通乡二级公路177公里。开工建设月照机场西南端盲降系统和8个停机位。开工建设1座中型水库、6座小型水库，建成2座中型水库。抓好民生保障项目建设，完成教育投资43.5亿元，医疗卫生投资24.5亿元。大力推动消费升级。深入实施扩大内需促进消费系列行动计划，有效扩大住房、旅游、养老健康、居民用电等消费。办好第四届“生态凉都?高山珍品”高原山地特色农产品展销会和第二届“凉都弥你红采摘”活动。

　　（五）全力以赴抓好改革开放重点任务。统筹推进重点领域改革。抓好省级“三变”改革试验区建设，出台农村土地“三权分置”实施办法，建立风险基金和农业发展基金，实现县区农村产权交易平台全覆盖。深入推进“放管服”改革。实现市县政府城市管理领域机构综合设置。加快国有企业改革。统筹推进资源配置、财税金融、生态文明等重点领域改革。全面提升开放创新水平。把扩大对东盟国家的开放作为对外开放的重点，加强与东南亚等国家经贸往来。积极参与酒博会、茶博会、数博会等重大活动。外贸进出口总额增长21%以上。引进500强企业4家，完成省内外招商引资项目实际到位资金1400亿元以上。

　　（六）全力以赴抓好生态建设和环境保护。加强生态建设。实施生态修复工程，提高造林质量，完成营造林100万亩以上，治理石漠化45平方公里、水土流失170平方公里。坚持铁腕治污。开展污染源普查排查，抓好电厂、钢铁、水泥等重点企业治污减排，加强三岔河、南盘江、北盘江重点流域综合整治，全面启动河长制，确保省控以上断面地表水水质优良率达到90%以上，城市集中式饮用水源水质、空气质量稳定改善。

　　（七）全力以赴抓好财税金融工作。管好用好财政资金。制定存量财源挖掘清单和新增税源清单。非税收入占比下降2个百分点。优化财政支出结构，严控一般性支出，严禁铺张浪费。加强沉淀资金清理盘活，积极争取转移支付支持。大力推广政府与社会资本合作模式。着力解决融资难融资贵问题。加强地方金融体系建设，推动浦发、光大、兴业等金融机构在我市设立分支机构，加快六枝、盘县、水城信用联社改制。鼓励金融机构围绕实体经济、小微企业、“三农”领域，量身定做金融产品，最大限度降低企业融资成本。继续分类别、分行业举办银企对接活动。加强政府债务风险防范。完善“借用管还”一体化机制，推动政府性投融资公司市场化转型，抓好高成本存量债务置换和消化。

　　（八）全力以赴抓好民生保障改善。推动教育优先发展。出台全市教育综合改革实施方案。全面实现“普十五”目标。盘县县域内义务教育基本均衡通过国家验收。创建省级示范性高中2所以上。加快建设健康凉都。建成凉都医院并实现开诊。实施基层医疗服务能力三年提升计划。扎实做好计划生育服务工作。广泛开展全民健身活动。抓实社会保障兜底。新增城镇就业9.46万人。进一步扩大社会保险覆盖面。建立健全特困人员救助供养制度。加强社会养老服务体系建设，提高基本养老金标准。维护社会和谐稳定。推进安全生产领域改革，深入开展两年安全生产强基固本攻坚，坚决防范和遏制重特大生产安全事故发生。开展食品安全示范市县乡“三级联创”。加强社会治理，深入推进“平安凉都”建设。

　　扎实办好“十件民生实事”。（1）压缩党政机关6%的行政经费用于教育精准扶贫。继续对建档立卡农村贫困家庭学生上高中、大学免除学杂费。在3个贫困县实施农村学前教育儿童营养改善计划。（2）力争公益性岗位安置就业困难人员6500人。将1486名建档立卡贫困人口转化为生态护林员。（3）农村、城市低保标准分别提高15%和10%。提高城乡居民基本医疗保险政府补助标准。建立县级以上公立医院预约挂号统一平台，乡镇卫生院远程医疗和规范化数字预防接种门诊全覆盖。（4）整合资源，优先保障贫困户入股产业、就业创业，实现4.62万户19.76万建档立卡贫困人口“三变”改革全覆盖并实现入股分红。（5）城镇保障性安居工程开工建设33540户，基本建成20000户。改造农村危房3000户。建设10个休闲园。（6）建设六枝特区新农村环境综合治理省级示范点。对3700个农户厕所实施无害化卫生改造。对4个以上乡镇实施消防应急救援力量及装备建设工程。（7）新建、改建农村公路860公里。实施农村公路安全生命防护工程，处置隐患里程247公里。（8）基本消除乡镇以上建成区移动网络盲点盲区，实现高速公路、高速铁路沿线有效信号全覆盖。新增130个行政村光纤网络全覆盖。（9）实施多彩贵州“广电云”户户用工程，新增用户29000户。新建乡镇广播影视综合服务站20个。新建、改造70个全民健身路径工程。建成34个村级综合文化服务中心示范点。农村电子商务示范县公共服务中心建设改造全覆盖，电子商务网点建设改造覆盖30%的行政村。（10）安装2500个公共安全监控视频探头，搭建视频图像云平台。重点公共区域安全视频监控覆盖率达到90%。

　　四、加强政府自身建设

　　面对新的形势、担当新的使命，我们将把政府自身建设摆在更加突出的位置，不断提高施政能力和服务水平，努力打造人民满意的政府。

　　（一）坚定不移全面从严治党。牢固树立“四个意识”特别是核心意识、看齐意识，坚决维护以习近平同志为核心的党中央权威，更加自觉地在思想上政治上行动上同以习近平同志为核心的党中央保持高度一致。认真贯彻党内政治生活若干准则、党内监督条例、党组工作条例等党内法规，严格落实政府党组全面从严治党主体责任，严守政治纪律和政治规矩，持之以恒贯彻中央八项规定和省委、市委十项规定。强化政府党组自身建设，落实双重组织生活制度，严格执行民主集中制，建立健全“三重一大”集体决策机制。完善政府权力运行监督机制，扎紧“制度铁笼”，打造“数据铁笼”，加强行政监察和审计监督，持续深化拓展民生监督。深入推进政府系统党风廉政建设，做到惩治腐败力度决不减弱、零容忍态度决不改变，全力营造风清气正的政治生态。

　　（二）坚定不移推进依法行政。依法全面履行政府职能，完善依法行政制度体系。健全完善政府法律顾问制度，定期清理和公布政府规章、规范性文件，做好规范性文件备案和审查。建立政府及部门重大行政决策程序制度、合法性审查制度和终身责任追究制度。全面推行“双随机、一公开”。严格按照法定权限和程序行使权力、履行职责，严格执法、公正执法、文明执法。大力推进“七五”普法。自觉接受人大及其常委会的法律监督、工作监督和政协的民主监督，加强与民主党派、工商联、无党派人士和各人民团体的沟通联系，进一步提高建议提案办理的落实率和满意率。完善政府议事规则和决策程序，全面推进政务公开，主动接受舆论监督和群众监督。

　　（三）坚定不移增强民生情怀。坚持以人民为中心的发展思想，牢记为民初心，推动政策向民生聚焦、服务向民生覆盖、资源向民生倾斜，把为民服务的成果“立”在六盘水大地上，“写”进群众心坎里。践行党的群众路线，多渠道了解社情民意，问政于民、问计于民、问需于民，深入群众开展调查研究，精准破解发展中的痛点难点，让发展实绩更有“温度”，让富民答卷更有“厚度”。坚持为民务实，多做雪中送炭、扶危济困的好事，多做标本兼治、打基础利长远的实事，严禁搞劳民伤财的“形象工程”“政绩工程”，严禁弄虚作假、虚报浮夸。大力推行“互联网+政务服务”，完善市县乡村全流程标准化大数据便民服务网上办事体系，实现政务服务城乡均等化，打通服务群众“最后一公里”。

　　（四）坚定不移履职尽责担当。坚持功成不必在我、功夫必须在我，以“开局就是决战、起步就是冲刺”的劲头，以“弱鸟先飞、滴水穿石”的精神，以“马上就办、办就办好”的作风，在战略部署上“扣扣子”，在责任履行上“担担子”，在工作落实上“钉钉子”，全力以赴推动各项决策部署落细落小落实。深化政府系统效能建设，强化政府工作人员业务培训，切实提高履职能力，坚决整治不作为、慢作为、乱作为。健全督查问效机制，建立完善重大事项、重要工作调度督办清单制度，挂图作战、挂牌督办，确保政府工作掷地有声、落地生根。完善正向激励和容错机制，为担当者担当、为干事者撑腰，让开拓进取谋发展蔚然成风。

　　各位代表，光荣的使命，鼓舞我们砥砺奋进；人民的期盼，鞭策我们勇往直前。让我们紧密团结在以习近平同志为核心的党中央周围，在市委的坚强领导下，不忘初心、继续前进，撸起袖子加油干，迈开步子加快赶，努力建设统筹城乡改革和发展先行市，奋力谱写六盘水新的精彩篇章，以实干实绩迎接党的十九大和省第十二次党代会胜利召开！
